# Society of Homœopathicians

**Published:** 1898-12

**Authors:** 


### SOCIETY OF HOMŒOPATHICIANS.

### MEETING OF 1898.

THE ORIENTAL, MANHATTAN BEACH, N. Y.,

TUESDAY, JUNE 28th, 1898.

FIRST DAY, AFTERNOON SESSION, 3 P. M.

Meeting called to order by the Secretary, Dr. Samuel A. Kimball, of Boston. Dr. Stuart Close, of the Borough of Brooklyn, New York City, was elected Chairman of the meeting.

Minutes of the last session were read and approved.

The reports of the Secretary, Dr. Kimball, and the Treasurer, Dr. Davis, were received and adopted.

The amendment of Dr. Thurston, striking out Sections 5 and 6 and the last four words of Section 4 of the Declaration of Principles, was adopted.

Dr. Wm. L. Morgan, of Baltimore, an applicant for membership, complying with a requirement of the By-Laws, read a paper before the meeting, entitled, "Some Morbific Agency Inimical to Life: What Is It?"

### BUREAU OF HOMŒOPATHIC PHILOSOPHY.

STUART CLOSE, M. D., Chairman.

After reading the Declaration of Principles and Rules for Practice the Chairman announced that the first paper to be read was from Dr. Pease, of Chicago.

It was moved and carried to invite guests to participate in the discussion of papers.

### PSYCHOLOGICAL HYGIENE.

BY FREDERICK O. PEASE, M. D., CHICAGO.

In taking up again in a related way the subject of my paper at our last meeting, in which was expressed my thought in regard to the causes of disease, I do not wish to seem persistent, nor is there intention to captiously force upon your attention my particular or personal views. I do desire discussion, and was disappointed in there being little last year upon this important subject. In these wonderful years of progress and achievement in every physical and spiritual dimension which man's moral and mental development can operate, there is manifested a grand desire to attain perfection. The measure of perfection attained always depends upon the measure of man's knowledge of the essentials of the particular *thing* he purposes to perfect.

We must first know and understand the causes of diseases before we can teach the laity how to avoid them, or how to curatively treat them ourselves. My conviction is that much of this knowledge is too often or exclusively relegated to the departments of hygiene and sanitary science, thereby being placed in a realm of study too much neglected by the active practitioner of homœopathics. There is much of value being developed in the domain of psychology and hypnotism also, which is going to waste because of the lack of comprehension of the essentials of existence or being by the otherwise keen mentalities that are investigating. An example of this going wrong is, their looking upon or following out the action or operations of the faculties and calling it *mind*, or soul, or the ego, not knowing that mind is a collective term, meaning the operation of the faculties, and not in any sense the faculties themselves or soul. Thus they go off into the realms of imagination and talk about the "duality of mind" or the "objective" and "subjective mind," etc., etc. But to return to my line of thought. So much depends upon our knowledge and its application of what is really hygienic and sanitary that the subject requires very broad handling, because there is, by rights, much to be included. They should not begin and end in fine calculations upon the cubical or square contents and air space in living and sleeping rooms, etc., nor upon the pounds and ounces of food ingested. The actual weight or thickness of wool or silk, linen or paper garments *may* have something to do in causing a fever or a melancholia, but there are other energies or essentials whose influence is being manifested.

It does not seem orderly or rational—*e. g.*, to depend upon artificial digestive ferments, whether or not they are "eneucleations," "distillations," or "isolations" of peptic or pancreatic juices or other physiological tissues and products from the great American hog, sheep, or steer. These are not "the one thing needful" to cure or restore a depraved digestion, appetite, or morbid function. The enormous "output" from the great slaughter houses, stock yards, creameries, etc., of these wonderful productions by the more wonderful and scientific "chefs" who manipulate so successfully the refuse of shambles is astonishing. Why! they can make a few pounds of pancreas or ounces of peptic glands reappear from their laboratories as some hundreds of two, four, or six-ounce bottles of digestives, enzymes, and metabolizers—with which to supply an army of smug sample peddlers who are eternally waylaying the profession. And the profession must encourage this, for the samples are always forthcoming year after year, in ever increasing numbers, while the dear public almost universally adopt and use them as panaceas or specifics for this, that, and the other real or imagined ailment. Also, *greater than all these*, the mass of prepared foods, mostly adulterated humbugs. All these are becoming a menace to health of great potential and miasmatic danger.

There must be a clearer understanding of the fundamentals of human life, in health and disease; a broader comprehension of the relationships between the functional activities and the physiological organs and tissues, and the essence which gives, by its presence, the life power to the body in which that essence moves and has its being. Then there will be clearer understanding of what *is* true hygiene and sanitation, when and how to use therapeutic measures that will go far towards reharmonizing the discordant elements of disease. The progressive physician will, in time, know these things, and he will have at heart the work of protecting health and teaching the people what should be avoided as disease-breeders, rather than adding to his bank account the proceeds of such fakes and fads. Pure air, plenty of space which is sanitarily clean, and orderly habits of eating, drinking, and sleeping are necessary for the conservation of health, as also are wholesome food and drink. But there is a more potent hygiene, a more saving sanitation even than these: that of the spiritual, moral, and emotional planes. It is from these that the potential energies and influences of the soul or essential man, through the power of life, manifests a protective, rejuvenating, and reconstructive office. From the higher planes of substantial and essential being must come the purification and correction of disturbed function and habits of the individual and corporeal man.

All processes of human life, or even of animal or vegetable life, are influenced from above downward—that is to say, from the higher planes of our essential and substantial being into the physical or material ultimation of that being. The identity and individuality of that essence, in generals and particulars, is expressed in physiological and anatomical forms, organs, and functions.

That influence, or rather that essence, if not interfered with, will proceed in orderly movement in accord with the laws and relationships governing the operation of both the essence and the physical home of that essence. Then there is equilibrium of energy, harmony, or health. If there *is* interference the previous orderly movement is broken, discord and even riot supervenes, and disease is the result. Such interference is always the beginning of punishment for broken laws.

I would have you understand that the *man* is not the body, nor is he the life, but he *is* the essence within the body, the strongest causative influence in the formation, growth, and *use* of that body, which is only a tent for a brief sojourn. The life is a quality of that essence, and the power of that life is brought to the cognizance of the bodily senses, and to our senses, through the material habiliment or ultimation of that essence. In this material habitation is assembled the members and instruments by which the man is enabled to operate by the power of life, obeying the laws which govern its sojourn here. We have then, in our being, in our consciousness of material existence upon the earth plane, two identities so harmonized as to operate as one; the essence, or soul, and its material complement the body. The harmonizing power, the uniting element which enables each of these identities always, at need, to exercise their separate functions, like a subtle fluid, permeates, vivifies, and propels the members of the body from the material side, and carries from the soul faculties to the bodily senses, or *vice versa,* this power of life. In an electric engine or motor is illustrated the process very nicely. The grouping of the various mechanical forms, connected together by the various wires and parts, according to established mechanical laws, and as the mechanic has formed or assembled them, we look at and pronounce it complete and perfect, and the maker a genius. But until the subtle electric fluid enters the mechanism it is *dead* matter, formed of the elements. The knowledge stored in the faculties of the essence, the man, the impulse to invent and to ultimate his impulse in form of mechanical grouping of instruments and members, each to perform its special function, shows or proves the existence of the essence; the machine, completed, is the material tent in which the impulse of genius is to live and move and have its being. The electric fluid is the power of life which is imparted to the machine, the body, and when actively engaged in its potential relationship between the maker and the made, between the essence and the impulse or purpose ultimated, evidence of *life* is demonstrated, the engine operates and performs the work or purpose for which it was formed. Thus we see the separate identities: Essence, or maker; engine, or body, electric fluid—the power of life. Not one could have being without the other. The essential power of life in man, the electric power of life in the engine *conjoins* the essence with the material, establishes harmonious activities, and energy proceeds through the whole from the essence. In either our engineer or in his engine everything must be in accord with the laws governing all things concerning the *being*.

In an attempt to define what life is we meet at once with some difficulty, because the word life is used in various senses, which leads to much confusion. It may mean a man's or tree's existence, or duration of an existence, or activities of a being, or cessation of being, the opposite of death. Emerson says: "Life is what man thinks and acts." Another philosopher says: "No, for if this were true, life is not worth the living." We speak of the life of a man, a tree, a building, or of a corporation, etc.

But what *is* life? Is it an entity, pure and simple? Is it a part of the body or of the soul? Is it an expression of energy alone, or is it parceled out to a man, a tree, or an oyster from some great reservoir in earth, air, or sky? Or does it proceed from the stars as the astrologer would have us believe, or from heaven itself? Thus we might continue making queries for all time, finding no answer unless we make use of the great faculties which are ours in abundance to enable us to comprehend, not only what life is, but the soul, its immortality, and more. It is not the province of this paper to demonstrate the soul, although *it is* logically and scientifically possible to demonstrate and prove the identity, individuality, immortality, and existence of the essence which has received the name soul. This is just as possible as it is to scientifically and actually demonstrate the existence of any one or all of the identities which have been classed as elements—gold, silver, sulphur, etc. Let me proceed to define life, then, that quality with which we as physicians have to deal.

In the first place, we must assume that the human body is the corporeal, visible, tangible home or tent within which the soul dwells. Therefore the body is not the man, the essence, the soul, any more than the eye is sight or the nose is smell. If the soul is immortal (we can prove that *it is*), and the body is not, then we must assume that the body was made for the soul, and as souls differ, so must the body which each soul occupies. Again, the body being corporeal, material (indeed, we know and can prove it is composed of thirty-three of the seventy-odd elements of earth) it cannot consist of or contain *per se* any of the intelligences, attributes, or faculties which belong to the soul. It is only an assembly of compounded and dull matter. We assume also that there is an essential difference between the attributes of this body of a man and those of a plant, tree, or stone. As an animal, man has faculties in number and quality which place him far above the other animal intelligences, and these are separate and distinct from the material molds which he or they occupy. These faculties being the qualities which qualify the essence which we have named soul, prove the existence of the soul essence, just as the material qualities, weight, density, ductility, etc., etc., qualify the essence of the material element, silver.

It is not difficult to conclude from many such obvious reasons that the difference between animal life and that of a plant or tree or metal is as wide as the difference between their material forms. Therefore, man, as to his essence, is clothed in the material form which that essence required, and which was a result of and ultimation of the essence. In the stages of development of the body, leading up to occupancy by the soul, it was presided over by material ultimations of its parental progenitors, but on the physical plane—after man the soul entered it, there was added to the parental influences his own as direct and additional formative influences. These more and more accentuated or modified the particular physical signs of parental influences until as a full-grown man he has his particular personality or individuality. The essence belongs to the spiritual realm, but, being on the material plane, is subject to the laws and forces of the material and unsubstantial world of forms for a season, and is destined in the succession of events to return to that realm, when his race is run.

We are now able to formulate this principle or law. Back of every object or form in existence, back of every identity, there is and must be an essence. Without an essence there cannot be an identity. Upon all sides we see, feel, and come in contact with the ultimations or media of the essences, but not with the essences themselves. We may not and cannot comprehend any one of these essences, but that is no reason why we may not know and prove their existence, for we always have our faculties with which to learn of and conate their qualities. The form in which an essence is embodied is the medium through which those qualities of the essence are manifested. We now have come to a point in our definition where I will advance the following postulate:

Life is a quality or attribute belonging to the soul which unites all the faculties of the soul together, that they may operate together in unison and affinity; it also connects the soul and its faculties with the external world, materially and spiritually; enables the soul to communicate by the faculties, each with the other, internally, and thence outwardly, and from externals to internals, all this in unison and affinity. That the soul may have material consciousness of material things the physical habitation is provided and furnished completely with all the members and instruments necessary, and so long as the habitation is in fit condition, it will be capable of manifesting the operation of the faculties of the soul.

Life is the quality which executes the wants and commands of the soul and acts through all the dimensions of relationships of the soul and body that there may be complete retroactive communications with all things within and around, materially and spiritually.

The wonderful mechanism, with all its special tissues and functions, its systems of rapid and effective accomplishment of orders, and fulfilling of the wants, etc., of the soul, is always conducted in accord with the laws of creative power. The special senses are the faculties of the material body—the nerves of sense, the means of telling the soul that sight, hearing, touching, etc., are being used. The organs of sense are but the special instruments through which the soul sees, hears, tastes, smells, and feels *material* things. These organs are not necessary for the spiritual senses. The eye is not sight, nor is the nose smell. The nerves form a sytem of highways and byways by which the *life* manifests power going to and fro, and through all the other systems over which the power of life acts in obeying the mandates of the ego which sits within its bodily kingdom. The bodily senses, functions, processes, chemical affinities, mechanical forces, and movements are for the operation of the power of life, and the preceding operations of those essential but physical ultimations of the spiritual essence all contribute to manifest the individuality of the soul.

Now we may begin to know and understand to what potentialities the body is subject; why sorrow and joy, disease and suffering must come if the soul develops along distorted lines, or departs so far from its spiritual and essentially clean uprightness as to devote its faculties and power of independence to a wrong use.

There can be no doubt or denial of a legion of disease manifestations that come directly and indirectly from using the faculties in a wrong and unwholesome direction. These, rightly understood, will prove the office of the power of life, for the phenomena of action and reaction, so much talked about by the homœopathician alone, is but evidence of the efforts to overcome the distuning effects of wrong living, wrong habits of emotional and mental activities.

Since we have to acknowledge the facts of mental therapeutics, why not the causative and distuning influence of mental states? If a patient's belief in the sophistries and imaginations of a mind curist, or in the psychical curios of the hypnotist, will lead him to resign his self-control of emotions and sensations, or his power of life to take orders from an outside essence, why should not there as surely be development of emotional, mental, and physical distortion and disease from within?

Hypnotic suggestion, Christian science, and all these psycho-therapeutic systems may be compounded together and shown to be only different expressions of different observers from their several view-points, of the manifestations of this one power of life.

We cannot deny that bodies inherit certain tendencies, but those tendencies only conern the physical conformations, family types of form or feature. In the body having *peculiar* tendencies which differ from the family types, there must be an essence of peculiar type of action. The peculiar actions or methods of that soul show that its tendencies must have been caused by past habits of action. A soul with a certain tendency will take birth, or choose to be born in a body which is the fittest instrument for the display of its powers or faculties. This must be so, if there is anything in the laws of affinity, which are only the result of habit, and habit is got by repetition. We are in the habit of thinking of ourselves as the body—*i. e.*, it is a fact in everybody's consciousness that he thinks of himself as the body, but how did he come to think of himself as matter? In essence the soul is not bound by matter. It is free, untrammeled, perfect, but while in the body it is bound by matter for a season and for a purpose. As the soul has the power of independence we must conclude that from choice it has taken up its abode in the body, and so there must be a purpose back of that choice. With knowledge of what that purpose is, and a desire to gain it, the soul will then master matter, make of the body a *servant*, and not be a servant of the body. We frequently see evidences of the soul's power upon the functions of the body, even though the body may be unconscious of it, or at least unable to prevent the action, as in the action of fear upon the sphincters, or upon the circulation and heart action, in shame or chagrin, and other like effects. These emotions are of the soul. They cause the material effects upon the organic functions

"Psychology is the science of soul forces and their action in controlling other people as well as one's self." A resolute will can often wake up and electrify the whole system, even when that system is under the languor or disease action of some other essential force. The nerve forces wake up, the blood courses more strongly, and new vigor is awakened, showing what an influence the soul faculty of will has over the power of life. But the will could not thus act unless by the combined aid of the other eight great faculties.

Psychology, the study of mental forces, acting upon and through the power of life, must become a large part of the medical man's work, and that study must be prosecuted in all the dimensions of the science—must embrace the spiritual plane of action of the essences as well as the material and physiological. There are dangers in the attempt to apply the principles of psychology, because many, from an imperfect knowledge of it, or imagining that they know psychology (*e. g.*, in the branch known as hypnotism), will use psycho-therapeutics contrary to sound reason. Or they may, in utter ignorance or unconsciousness, act upon impulse and be drawn to the marriage altar, mistaking the spell which each has thrown over the other for true conjugal union, when it is only an expression of physical instincts. Parents who are trained in some medical or religious or political rut rear their children in the same narrow channels. It must be remembered also that one may throw a strong influence over another without the least desire of so doing, or without any wrong intention.

I pause here, and will not make the application of these principles in illustrating the causative influence of the spiritual forces upon disease. My paper last year should have followed this one. Both are imperfect enough, for this is a vast field for study, discussion, and wise experiment. I hope that there is food for thought, and that it is not offered in so bungling a manner as to thwart my desire to add interest and profit to our meeting.

### Discussion.

Dr. Kennedy—I realize that when we have a paper of this kind, bearing upon homœopathic philosophy, we are dealing with something that is very difficult. I have been very much interested in this paper by Dr. Pease. I admire his courage, Mr. Chairman, in grappling with the problem. I suppose it may seem to some that the time spent in attempting to handle a subject of this sort is time lost, but I do not sympathize with that point of view. We have in this bureau of homœopathic philosophy our most interesting papers, at least to some of us. I believe it is right to spend sufficient time to put on paper our thoughts relative to this department of science, with which we, more especially in our branch of the profession, attempt to wrestle.

I feel that in order to properly discuss a paper of this sort we should have an opportunity, as members of the society, to read the paper carefully before it is presented, in order that we may digest it, and so formulate our thoughts that they may be expressed more intelligently.

The essayist spoke of this "essence," this "internal essence" as being the man; and I mention it, because our friends of the mind cure make this strong claim, that the man is not the body, is not material, but is the mind. Now, I have no idea, Mr. Chairman, that our essayist intended to convey any such idea. I suppose he meant that the essence is the *essential* man, for it seems to me the essence within is not the man, the material is not the man, *no one part* is the man, but the whole together, the beautiful harmony, is the man. That is the broad sense, the only sense in which we can deal with him—the whole together. I have no doubt, Mr. Chairman, that our essayist will clear that up, if it needs any explanation.

Dr. Patch—I feel the utter impossibility of discussing a paper of this kind without having previously considered the subject. The question is a very important one, but I must confess my mental state after listening to Dr. Pease to be one of confusion. I do not know that anything is gained by going into the minutiæ of these points. Life is a state rather than a power or force. The latter term does not seem to convey a clear idea of life to one's mind, and the state of the whole man would seem to constitute life more than any particular action of the soul or so-called "essence" of the man. The vital force, as we understand it from Hahnemann, would seem to be an intermediary, between material and spiritual elements, rather than the true life power. Then, too, in the beginning of the paper, it seems to me that there is confusion in the use of the term hygiene and the products of the laboratory. I think we, as homœopathists should enforce the laws of hygiene as far as they are in harmony with our beliefs. I believe that true hygiene is always in harmony with Homœopathy, but the use of the laboratory remedies and fads is not hygiene. It may be claimed as hygiene, but it is only a makeshift of those who have no further resource.

Dr. Morgan—I can hardly enlarge on this on general principles. I would, however, like to hear some opinion in regard to the great variety of life forces, and the essential properties of life force in very different material substances, and also in regard to the action of those different life forces in disease. Also regarding nourishment, and whether the natural life requires continual nourishment, something that the chemist does not know anything about.

Dr. Rushmore—In regard to the essential constitution of man, I seemed to get from the paper the thought of dual rather than triple constitution. I think we are bound to consider ourselves as consisting of three parts. The view of a triple constitution is given in Scripture. It is spoken of there as three, the body, soul, and spirit, man not being complete without all three of these, and man finds his activity and expression in all three of these. Another thought—it would seem to be the trend of the paper that the soul is always predominating. Do our material remedies taken into the body change the state of the soul? I simply throw that out for consideration. I do not oppose the thought in the paper, but I will instance the action of gold upon a suicidal tendency. It affects the soul, and the mind becomes cheerful. That which acts primarily on the body acts secondly on the soul.

Dr. Kennedy—I want to emphasize what Dr. Rushmore has just said. We all recognize the fact that remedies will do what Dr. Rushmore has just said they will do. Dr. Rushmore has said that when Aurum is administered to a patient threatening suicide, it will change the mind of that patient so that he will desist, and will become cheerful. He will become really another person. I want to emphasize what I believe to be true, that when the remedy is given potentized it has that effect; not to imply by this that any of us present use remedies that are not potentized, but I believe that the higher potencies are more likely to act in this way. I am not prepared to say that the lower potencies will not do this, but I believe we can place greater reliance upon remedies more highly potentized—I mean a remedy not under the 200th. They seem to touch that innermost life. They become possessed, so to speak, of that life force which is akin to that inner man which we as homœopathists know we can reach.

Dr. Davis—I would like to ask Dr. Rushmore if I correctly understood him to use the word "material;" did he say "material remedies?"

Dr. Rushmore—I think so.

Dr. Davis—That raises a point as to whether a material substance acts in the sphere of the spiritual except as a vehicle of the dynamic force. If it were so our higher potencies would not act as well as our lower. The nearer we bring potencies to a similarity of plane, the more perfect is the result, as has been referred to. I do not know that I understood the doctor correctly in his remarks.

I understood the writer to say that he considered vegetable life or processes and human life as far apart as the poles. That may be so, but I never like to express it in that way. If this is true it would debar vegetable remedies from acting upon man. It can hardly be possible that they are as far apart as we suppose.

Dr. Pease—I suppose I said that they differed as their forms differed.

Dr. Davis—I think it has been said that they are very similar, so there is a mistake somewhere.

Dr. Rushmore—I think we must hold to the material nature of the remedies that we use, otherwise we come into the domain of the mind curists. The remedy must be put into the patient's body. The fact that it becomes invisible does not demonstrate that it ceases to be matter recognizable by tests.

Dr. Close—It is a sad thing for Homœopathy if its exponents are obliged to take the position of materialists in order to combat the position of the metaphysical healers. We have only to make a very brief inquiry into *The Organon* to find that Dr. Rushmore's idea will not find any endorsement there. Hahnemann opposes all who hold to the materialistic theory. He reiterates and emphasizes with the greatest force the idea that disease is dynamic, and that remedies are essentially dynamic. The remedy corresponds to the disease, and is similar. It has the power to produce a corresponding state of the system.

Dr. Pease evidently uses the word "essence" in the sense that "spirit" is commonly used. It ought not to take any more argument to convince thinking men of the existence of spirit or "essence" than it does to convince them of the existence of matter. As a matter of fact you cannot by argument prove the existence of either, because both are perceived intuitively by the mind. We all hold practically the same view, but there is confusion in the use of terms. Matter cannot cease to be, though it may change its forms. Spirit or essence is eternal, but it is eternally manifesting and individualizing itself in and through matter. Spirit and matter are in reality one, for neither could exist without the other. They are the two sides or phases of the same thing—the upper and the under, the inner and the outer. It is impossible to mark the point where matter becomes spirit or spirit becomes matter, but there is an eternal flux or interaction between the two. With that in mind the infinity of individualized forms ought not to confuse us.

Dr. Patch—It seems to me that Hahnemann uses the term "spirit-like" rather than "spirit" in speaking of remedies, and in speaking of diseases he uses the term "dynamic." If that word implies a spirit-like form of remedy, such remedy may still remain essentially material. It is an open question whether the raising of the potency of a drug changes its degree from one plane to another. I do not think it does.

Dr. Kennedy—This question of matter, it seems to me, is very fine; it turns upon what constitutes matter. I felt while Dr. Rushmore was speaking that he was right. I still feel that way. I do not think that Hahnemann's idea is different.

I think that Hahnemann, in using the word "material" used it at that time (over 100 years ago) in the sense in which the word was employed in those days, as over against that form of remedy which he taught was the best, and the action of which he called dynamic and spirit-like, as Dr. Patch has said. To come back to the question which turns upon "What constitutes matter?" What constituted matter in the days of Hahnemann would not be recognized as constituting matter to-day, for our ability to determine matter has become magnified, and that which in the days of Hahnemann would have been considered fanciful and beyond the realm of matter is now demonstrated by scientific research, by means of the microscopical and chemical laboratories. Substances which in those days were not recognized are now brought forth and understood by man. As I said before, the whole question turns upon what constitutes matter. We have not yet the ability to determine matter from a scientific point of view. That which to-day seems to be beyond the realm of matter may, in another century, be demonstrated by processes which will be accepted by those who will be the materialists of that time, so I think we had better go carefully along this line.

Dr. Adams—I have been very much interested in Dr. Pease's paper and the remarks that have followed. It seems to me that to Hahnemannian physicians the study of what the vital force is, is of the utmost importance. It is a vast subject. Whether we can come to a decision or not is very problematical to me. It occurs to me that such studies should teach us to avoid dogmatism. Hahnemann has given us a law which we believe to be a law of nature. We have been applying it in a material manner, but we do know that cures are made by mental scientists. I see cases every week that are cured. I think we should approach these men with humility, not with our minds made up that there is nothing in their claims. We do not know how the potency is given by the mental healers, but it does seem as though we were yet coming to a higher potentization.

Dr. Pease—Mr. Chairman, I see that in my paper as I read it I have not accomplished what I wanted, because in my fear of taking up too much time I have cut out a good deal, thinking the minds here were posted, and would understand intuitively the position I have tried to make clear, but I see that I have failed to make certain important points clear. In regard to our friends, the mind curists, whom Dr. Kennedy mentioned in his first remarks, and of whom Dr. Adams has also just spoken, we know that we must acknowledge facts which they demonstrate. We also must acknowledge the fact that almost all of the various systems of psychological therapeutics do perform cures. Why they succeed or how they do it, is not a question to discuss here, but there is one point we must not lose sight of. I cited it in my paper—that is, back of every form we meet in nature or about us there must be an essence. If we keep that in mind we will not be confused when we talk about what material is. If we look upon our remedies as materials, or material substances, or forms, with no essence back of that form, what is to hinder our making a very close imitation of that medicinal principle; for instance, of that plant? I look upon the material forms as simply clothing in which an essence is presented to our senses. The property that produces the curative influence is the identity of the essence back of the form. Considering gold as an illustration, whether that gold was given in the lowest potency of the first form of gold leaf, or of the golden nugget from the soil, it is the essence, the identity, the individuality which induces the suicidal tendencies, it is not the material form. In Dr. Close's remarks concerning Hahnemann's statements he must not forget Hahnemann's position in regard to the spirit-like, dynamic force. We cannot comprehend the essence, but we can demonstrate the existence of that essence by proving the qualities which qualify it. We as homœopathic physicians prove it every day, by every cure that we bring about, and that cure is the result of an essence. The homœopathic law of cure is an essence, and Hahnemann and every one of his followers but manifest the power of that essence. The 1,000th and 100,000th potencies are only different expressions or manifestations of the essence of the drug. Some one, I believe it was Dr. Patch, spoke of life as being "a state of being." We must not forget that words are simply vehicles upon which we load a thought, and that word "life" is a much-abused vehicle. The word "life" is used in many senses, and confusion results. We can use the word "life" in the sense Dr. Patch used it, "a state of being," the life the man leads, a good life or a wicked life. I tried in my paper to define the sense in which I hope we will use it—the sense of quality, or power, or force. In that sense life cannot be a state of being, because life cannot be the motive. If life is a state of being it cannot be the motive back of that state. Back of every life in nature there must be an essence, because without that essence there cannot be an identity. In regard to another use of the word "life," Dr. Davis understood me to say of the life of a tree or a vegetable, that the difference was as wide as the poles between vegetable and man. There is a vast difference between the life of a plant and the life of a man, and that difference is found in the essence back of the identity. The essence of Belladona is expressed to our senses in its visible forms, and in its method of activity upon the human organism. Just so the forms of all vegetable material differ according to the essence. Of all animal forms of life man is the highest. Back of every animal life is an essence and an intelligence; back of every vegetable form of life is an essence, but not an intelligence. A man or animal with intelligence moves itself to its food, or away from that which is uncongenial to it, or from one place to another.

Dr. Close—So does a plant, or a tree, sending out its roots toward water and its leaves toward the sun.

Dr. Pease—No! no! can a plant save itself from a fire? No. In order to be understood I must go back to our essence again. That which we call the life of a plant or a tree has no intelligence back of it. The power which we call life in a plant we must call some other name. A plant or tree has the power of growth, and it has this power because of chemical affinity and mechanical operation. The power that we call life in a man has intelligence back of it, but the growth of his body is the same as that of the plant. In other words, the growth of a man's body is from chemical affinity and mechanical operation, but it has a different thing back of it which I am trying to define in my paper. It is that quality which unites the faculties of the soul, or the dynamis, if you wish to call it so, together with each other, and with the body, so that they may get in unison and affinity.

What is growth? Matter grows by chemical affinity and mechanical operation by addition of more matter, and that matter, whether plant or tree, is derived from the earth and from the atmosphere, directed by the power of life. Growth goes on the same way in the human body, but the difference is that the human or animal body has this intelligence back of it, while in the plant it is absent. A plant may be growing near a stream. If we dig down we will find that its roots are growing toward the stream until they reach the moisture. Does that show intelligence? No; it shows mechanical operation and chemical affinity. There is no intelligence there.

Dr. Rushmore spoke as though he understood me to object to, or at least to have forgotten to allow for the existence of a tripod type. I do not mean to be understood that way, but in order to understand each other we would have to go back to the absolute meaning of the words, because there is a difference in the meaning between the soul and spirit. I did speak of a tripod type, and a power of life which unites the three. One question I would like to ask, How can a material force, if that is the correct term, change the soul? The instance was made of gold changing the soul from a suicidal tendency to some other condition which was not suicidal. Was that suicidal tendency a part of the soul, or was it only a manifestation of changed conditions, or a departure from the normal, essential condition? There can be no doubt that a suicidal tendency is a sign of disease, and has the essence back of it. It arises from a wrong use of the faculties, and not from the diseased tissues of the body. What is it that gives individuality or identity to our Aurum, or to Belladona, or Silver? I claim that it is that essence which is inherent in every substance about us, and that the substances differ according to that essence, and hence there must be differing material forms which manifest them.

